# “A Group of Fellow Travellers Who Understand”: Interviews With Autistic People About Post-diagnostic Peer Support in Adulthood

**DOI:** 10.3389/fpsyg.2022.831628

**Published:** 2022-03-07

**Authors:** Catherine J. Crompton, Sonny Hallett, Christine McAuliffe, Andrew C. Stanfield, Sue Fletcher-Watson

**Affiliations:** ^1^Patrick Wild Centre, Division of Psychiatry, Centre for Clinical Brain Sciences, University of Edinburgh, Edinburgh, United Kingdom; ^2^Salvesen Mindroom Research Centre, University of Edinburgh, Edinburgh, United Kingdom; ^3^The Autistic Mutual Aid Society Edinburgh, Edinburgh, United Kingdom; ^4^Department of Psychology, University of Stirling, Stirling, United Kingdom

**Keywords:** autism, diagnosis, mental health, peer support, post-diagnostic support

## Abstract

Receiving a diagnosis of autism in adulthood can be a life changing event, impacting identity, relationships, and mental health. A lack of post-diagnostic support has been highlighted by autistic adults, their allies, clinicians, and service providers. It can be a source of distress for autistic adults, reinforcing feelings of social isolation and rejection. Peer support could be a cost-effective, flexible, and sustainable model to provide community-based support for autistic adults. However, there is little research on the value of peer support, despite calls from the autistic community. This qualitative study explored autistic experiences and needs post-diagnosis, identifying specific ways that peer support may benefit them, and exploring the limitations of peer support. Twelve autistic adults who had all received an autism diagnosis in adulthood completed a semi-structured interview focussing on the diagnostic experience, post-diagnostic support needed and provided, engagement with the autistic community, and post-diagnostic peer support. Thematic analysis of interview transcripts resulted in four themes: (1) Mismatch in support needed and provided; (2) Community connection; (3) Flexible and personalised support; and (4) Sustainability. Participants indicated that peer support may be a useful mechanism to support autistic adults’ post-diagnosis and offers unique opportunities not available through other support channels. Though informal peer support exists, it could be more sustainable and effective if well-supported and funded.

## Introduction

Due to broader diagnostic criteria and increased public awareness (Rutter, 2005; Hansen et al., 2015) many autism diagnoses now occur in adulthood (Fombonne, 2005; Happé et al., 2016). People seeking a diagnosis of autism for the first time in adulthood may diverge from prevailing stereotypes of autism. For example, they are less likely to be male (Gould and Ashton-Smith, 2011; Bargiela et al., 2016) and less likely to have a learning disability than those who receive an earlier diagnosis (Matson and Shoemaker, 2009; Geurts and Jansen, 2012).

Though autism diagnoses are more widely available, there are many barriers to efficient diagnostic assessment pathways, and appropriate post-diagnostic support (Huang et al., 2020). The adult diagnostic process involves standardised measures, alongside clinical interviews requiring knowledge of developmental milestones, and reflecting on challenges and difficulties throughout life (Rutherford et al., 2016a). Difficulties with the diagnostic process, including long waiting lists, multiple referrals, and complex routes to diagnosis (Jones et al., 2014) are well documented (Crane et al., 2018). Moreover, atypical communication styles and social anxiety make reporting symptoms difficult (Murphy et al., 2016). Concurrently, clinicians face an increasing number of referrals and constraints on resources (Rutherford et al., 2016b).

The lack of post-diagnostic support has been highlighted by autistic adults, the families of autistic people, clinicians, and service providers (Griffith et al., 2012; Lewis, 2017; Crane et al., 2018; Raymond-Barker et al., 2018; Huang et al., 2020). Most adults receiving an autism diagnosis are dissatisfied with the availability and quality of post-diagnostic support available, and many autistic adults report that they are offered no support whatsoever (Jones et al., 2014). There can be an assumption that because someone has managed to reach adulthood without support, that no support is needed (Griffith et al., 2012). Some receive only written information, which is considered inadequate (Beresford et al., 2019), and many find that the autism service they are referred to does not meet their needs, as support is aimed at children or those with intellectual disability and/or language delay (Griffith et al., 2012; Crane et al., 2018). Due to a lack of resources, clinicians are often unable to provide post-diagnostic support, leaving autistic adults disappointed with the diagnostic process and outcome (Jones et al., 2014; Crane et al., 2018; Huang et al., 2020). Post-diagnostic support not being offered or available is a source of distress for autistic adults, reinforcing feelings of social isolation and rejection (Beresford et al., 2019). The types of post-diagnostic support most frequently requested by autistic adults are support groups, social skills training, and counselling (Jones et al., 2014), with an individualised approach to support highlighted as enhancing independence (Griffith et al., 2012; Huang et al., 2020).

### The Impact of Diagnosis

Receiving an autism diagnosis as an adult can be a life changing event (Arnold et al., 2020). Though most autistic adults report feeling relieved, many also feel anxious, confused, upset, or angry (Jones et al., 2014). While diagnosis can increase self-acceptance and self-understanding, it does not necessarily improve acceptance or understanding from others (Punshon et al., 2009; Crane et al., 2018; Arnold et al., 2020). Previous research has found that long-term partners report initially reacting to diagnosis with anger and hopelessness, before accepting and supporting their partner (Lewis, 2017). Autistic adults report some negative reactions from their parents (Crane et al., 2018), and even when family members accept their diagnosis, effective support from non-autistic family members is hindered by a lack of understanding about autism (Punshon et al., 2009; Crane et al., 2018).

Diagnosis can have a profound impact on identity (Punshon et al., 2009), and some autistic adults have described how identifying as autistic opened up access to a community of autistic people where they felt they fitted in Punshon et al. (2009). Being part of a community can help in developing a sense of acceptance and pride (Davies, 1996), and thus engagement with other autistic people may be beneficial (Skirrow and Farrington, 2008; Punshon et al., 2009). Recent research has found specifically that self-acceptance and pride in being neurodivergent (Milton and Sims, 2016; Gillespie-Lynch et al., 2017) is linked to lower depression scores (Cage et al., 2018), and higher self-esteem (Corden et al., 2021) and feeling part of an autistic community reduces suicide risk (Cassidy et al., 2018). There has been a call for research that identifies ways of promoting the development of a positive autistic identity following a diagnosis in adulthood (Corden et al., 2021; Maitland et al., 2021).

While autistic/non-autistic interactions can be positive (e.g., Smith et al., 2021), autistic adults have often described interacting with other autistic people as more comfortable, validating, and fulfilling than interacting with non-autistic people (Bargiela et al., 2016; Hickey et al., 2018; Tan, 2018; Crompton et al., 2020a). There may be an ease of interaction with other autistic people that is not experienced during interactions with non-autistic people, as difference is normalised, and normative expectations of communication style do not apply (Bargiela et al., 2016; Hickey et al., 2018; Tan, 2018; Crompton et al., 2020a). With other autistic people, there is less need to conceal overtly autistic behaviours such as stimming and rocking (Crompton et al., 2020a). Engaging with this new social world can encourage self-compassion, build resilience, and develop a greater sense of autonomy and agency (Crompton et al., 2020a; Leedham et al., 2020). Recent empirical research suggests that interacting with other autistic people might be easier and more comfortable than interacting with non-autistic people, and that autistic people may have particular communication styles that are enhanced in autism-specific social interactions (Heasman and Gillespie, 2019; Crompton et al., 2020b,c; Rifai et al., 2021). Additionally, autistic adults have high levels of scientifically-based knowledge of autism and lower stigma toward autism than non-autistic people (Gillespie-Lynch et al., 2017). As formal post-diagnostic care for autistic adults is lacking, social relationships can offer valuable support, with support from autistic peers considered as particularly important. Thus, it is pertinent to examine community-based peer-support interventions that enhance autistic wellbeing during the post-diagnostic period.

### Peer Support as a Community-Based Post-diagnostic Intervention

The peer support model assumes that shared experience of a phenomenon enhances the development of an empathetic supportive relationship (Repper and Carter, 2011). In non-autistic groups, peer support yields substantial mental health benefits for recipient and provider (Repper and Carter, 2011), and is easily implemented within existing services whilst providing positive benefit at lower cost (Pfeiffer et al., 2011). Peer support structures can take several forms, such as mentoring, befriending, and support groups (Bradley, 2016). Mentoring, in particular, has been shown to have several benefits, including the provision of a cost-effective personalised supplement to formal support mechanisms, the promotion of rapport due to proximity in age, and increasing the wellbeing of both the mentor and the mentee, with the latter continuing to experience benefits a year later (Jacobi, 1991; MacLeod, 2010; Appel, 2011; Hillier et al., 2019). Despite its popularity, the concept of mentorship is ill-defined and many definitions exist in relation to particular subject areas (Crisp and Cruz, 2009). The most widely accepted definition relates to the formation of a goal-oriented supportive relationship in which one partner is more experienced and offers guidance to the other partner (Jacobi, 1991). Autistic peer support is an opportunity to provide support for autistic people in an accessible way, which embodies their priorities, and unlike many other supports does not involve the centring of a neurotypical lens (MacLeod, 2010; Stevenson et al., 2016; Thompson et al., 2020).

### Autism and Peer Support – The Evidence to Date

A small number of studies have examined autism-specific peer support in adults. Most of these studies have involved support delivered through online support groups (MacLeod, 2010; Stevenson et al., 2016). MacLeod (2010) details the creation of an online support network for autistic university students, the AS portal. Discussions were started by moderators and by participants, with the latter evoking a more sustained response and engagement. Although the number of students who participated was relatively small (*n* = 7), the success of the participant-led discussions was interpreted by the authors as illustrating the willingness of the students to engage with one another and with this form of support. Participants highlighted that they appreciated the opportunity to offer advice and support to others. MacLeod (2010) considered the willingness and engagement of autistic people in these networks to be a “vast untapped resource” and highlighted the potential of online peer support networks as a mechanism to raise “collective consciousness and personal self-confidence” (p. 23). Rosqvist (2018) used focus groups (*n* = 7) to explore the topic of autistic peer support with a group of autistic adults who worked with and provided support to young autistic adults in an autistic-only workplace. They sought to outline an alternative model of autistic development, underpinned by common experiences, mutual understanding, and a focus on ways of being that are different, rather than deficient. In Martin’s et al. (2017) development and evaluation of a training programme for mentors of autistic adults (*n* = 9 pairs), the importance of supervision and ongoing training was emphasised, as well as reliability and consistency on the part of the mentor. Although the majority of the mentors in this study were not autistic, those in same-neurotype pairings reported benefitting from the enhanced empathetic closeness brought about by similarity of experiences between the mentor and mentee. Finally, Crane’s et al. (2020) study involved sixteen participants engaging in 10-week autistic-led programme that aimed to support autistic adults to learn more about autism within a peer group context. This was designed as a post-identification course for people who recently identified as autistic or who had recently received a diagnosis of autism. This study demonstrated the importance of valuing autistic individuals as “experts by experience” and the egalitarian potential of peer support in terms of deconstructing the hierarchy between predominantly non-autistic professionals and autistic individuals.

### The Current Study

While further research examining the efficacy of a post-diagnostic peer support intervention for autistic adults is needed, a crucial first step is to ensure any peer intervention is co-designed with or led by autistic people and reflects their views, preferences, and priorities. The purpose of this study was to elicit the views of people who had received a diagnosis of autism in adulthood, exploring their diagnostic experiences, the post-diagnostic support that they needed and were provided. We focussed on what the function and focus of peer support may be, whether they felt it could act in place of other support, should exist alongside it, or not at all, and the benefits and challenges that they suggest it may face. In this study, we used a qualitative methodology to examine these views and explore how these may be incorporated into a future support system.

## Materials and Methods

### Methodological Approach

This study used a qualitative design, with semi-structured interviews analysed thematically. Ethical approval was obtained from the University of Edinburgh Moray House School of Education and Sport Research Ethics Committee.

### Participants

Participants were 12 autistic adults (see Table 1 for demographic information). Participants were recruited through our project website, local autism organisations, and social media. The project advert was titled “post-diagnostic support and peer engagement,” and participants were told they would be asked questions about their diagnosis, the support (or lack of) received after diagnosis, and social relationships and support from autistic and non-autistic people. Participants were eligible if they received a formal diagnosis of autism (including Asperger’s syndrome and other “on the spectrum” diagnoses) after the age of 18, spoke English to a native level, and had received their diagnosis within the last 10 years. We asked that participants had received their diagnosis within the last 10 years to ensure that the diagnostic experience was recent enough that participants could reflect on their experiences and needs at that time. A limit of 10 years allowed a range of experiences to be represented, while also ensuring that experiences were representative of recent and current practices in autism diagnostic pathways.

**TABLE 1:** Participant demographic information.

ID	Gender	Age	Age at diagnosis	Years since diagnosis	AQ score
1	Female	54	47	7	49
2	Non-binary	48	44	4	37
3	Female	31	21	10	39
4	Male	63	61	2	47
5	Male	50	48	2	39
6	Female	30	29	2	39
7	Female	43	41	2	43
8	Female	46	43	3	40
9	Male	36	30	6	42
10	Female	33	30	3	37
11	Female	39	37	2	43
12	Male	66	58	8	40

*AQ, autism quotient.*

Participants (7F/1NB/4M) had a mean age of 44.92 years [standard deviation (SD) = 11.94], and a mean, autism quotient (AQ) score of 41.25 (SD = 3.74). The mean age of diagnosis was 40.75 years (SD = 12.01), and mean time since diagnosis was 4.25 years (SD = 2.8). All participants identified as white British, Scottish, or European. A number code was generated for each participant and identifying details redacted from reported quotes.

### Procedure

All participants provided written informed consent before the study commenced. Before the interview commenced, participants were told (1) that they could take a break at any time during the session for any reason, (2) they did not have to talk about anything they did not want to talk about, and (3) if they wanted to answer a question in more detail, they could go back to a question or answer it in more detail. The first author conducted interviews either in-person (*n* = 2), over the phone (*n* = 1) or via video call (*n* = 9) depending on the preference of the participant.

### Measures

#### Semi-Structured Interview

Qualitative data were collected using a bespoke semi-structured interview designed for this study in consultation with autistic collaborators. Using a semi-structured approach, the interviewer can probe a participant’s response and gain clarity where there is ambiguity (Barriball and While, 1994). The wording of questions was designed to be neutral and not leading, and was reviewed by two autistic people prior to the study commencing to ensure that the language was accessible and comprehensive. The interview questions explored the diagnostic experience, post-diagnostic support needed and provided, engagement with the autistic community, and post-diagnostic peer support (see Supplementary Table 1).

#### The Autism Quotient

The AQ is a 50-item multiple choice questionnaire which yields an approximate measure of autistic traits (Baron-Cohen et al., 2001), and was included to provide additional detail about participants’ autistic traits. This was completed individually by participants, using an online form, following the interview. A score over 32 indicates a high level of autistic traits.

#### Qualitative Data Analysis

Interviews were transcribed by a professional service and checked for accuracy by the first author. The six-phase framework of thematic analysis was then applied (Braun and Clarke, 2006). This method of analysis allows an inductive process: it does not necessarily rely on existing frameworks for interpreting data, and thus it is suitable for an emerging area of research such as this (Willig, 2013). The six-step process involves familiarisation with the data through reading and re-reading interview transcripts, generating codes that highlight pertinent features of the data, searching for themes, ensuring themes relate back to the initial codes, defining and naming themes, and relating the findings back to the research literature (Braun and Clarke, 2006). The analysis was led by the first and second authors. The first author completed initial coding of the data, and these were then discussed with the second author. A broadly inductive approach to analysis was taken; data were coded by category, and the similarities and contrasts between participant responses were examined using a constant comparative method (Glaser and Strauss, 1967; Miles and Huberman, 1994). In collaboration, the first and second author then searched for sub-themes and themes, reviewed these themes, and defined and named the themes. Establishing themes was a data driven process rather than attempting to work within a pre-conceived coding framework (Saldaña, 2021), though our knowledge and experience of the literature may have shaped the analysis phase (Braun and Clarke, 2021). The first author is a non-autistic researcher, and the second author is an autistic adult with a background in advocacy: thus the views of mainstream psychological theory and the lived experience perspective of autistic adults are incorporated in this analysis.

## Results

**FIGURE 1:**
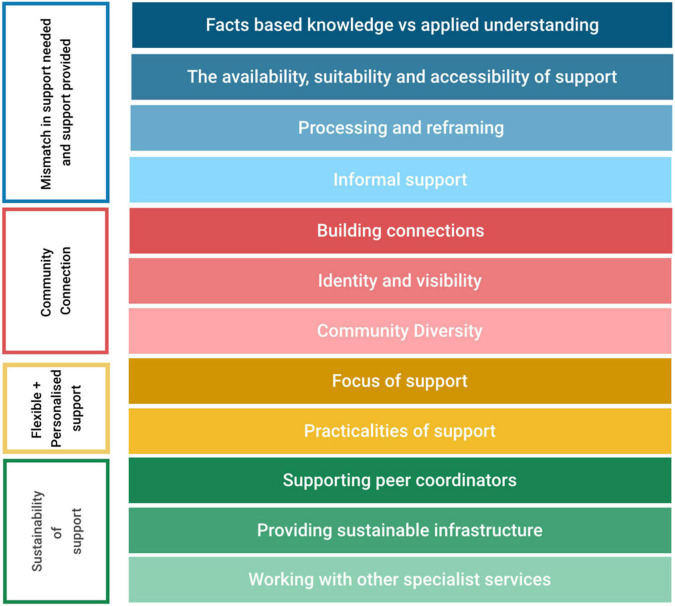
Structure of themes and subthemes.

Participants reflected on their experience of diagnosis, their support needs around that time, and their experiences interacting with autistic and non-autistic peers. Four main themes were identified and 12 subthemes were identified from the interview data (Figure 1). Participants quotations are reported along with their gender and age in brackets.

### Theme 1: Mismatch in Support Needed and Support Provided

The first theme focussed on the mismatch that participants experienced between the support they needed and the support they received following their diagnosis. Participants felt that the support they received (if any was received at all) did not meet their needs. They stated that support should focus on enhancing understanding on how this diagnosis applied to their lives, their relationships, and future plans, and that support should come from sources that understood their experiences.

#### Subtheme 1: Facts-Based Knowledge vs Applied Understanding

The information provided post-diagnosis was mostly in written form and described what autism is, which participants found insufficient and not helpful. The content of written resources focussed on facts about autism, whereas participants wanted to know what being autistic meant/looked like for them, and what it meant for their relationships and identity.

*“I got a print out leaflet about Asperger’s. They weren’t particularly useful suggestions. I already knew about autism cause I had studied it, and I had worked with autistic kids, so that wasn’t much use to me”* – Participant 3 (F, 31).

The personalised information that participants received in their diagnostic report was often framed in a very negative and pathologising way, and was difficult to process. “*Reading the report, it is written in quite a negative way. Because it is the deficit model. So it was quite hard reading the things about myself that I am bad at. Cause I have tried my best to hide them, for the last 40 years. [*…*] I am an intelligent person, I am a very capable person but [*…*] this new diagnosis and this new information [*…*] I was still not quite sure what to do with it*.” – Participant 11 (F, 39). Some participants described the negative impact this had on their self-esteem – *“the diagnosis had a knock-on effect on my confidence at that time [*…*] I think I needed probably a bit of help to process some of that stuff.*” – Participant 6 (F, 30).

Participants had a good understanding of autism and its core diagnostic features in a general sense, but had difficulty understanding what this meant for them on an individual level. As autism is very heterogeneous, many struggled to identify with the information provided to them. What most participants wanted was the opportunity to discuss what the diagnosis meant to them.

*“There wasn’t any help. You’ve just been diagnosed with something which is a neurodevelopmental condition and a disability and you have had it all your life, it was quite*… *well it was frustrating but it also it was quite*… *in a way quite scary to be sort of*… *left unsure how to manage this in*… *from the rest of my life going forward”* – Participant 5 (M, 50).

#### Subtheme 2: The Availability, Suitability, and Accessibility of Post-diagnostic Support

Many participants reported requesting post-diagnostic support and being refused, or told none was available.

*“I mean there is nothing available really [*…*] I did go to my GP a few months after my diagnosis and explained*…*but they couldn’t help me. My GP was quite understanding*… *really understanding, really, and put me in touch with somebody fairly senior from the local adult autism team*… *but they weren’t very helpful.” –* Participant 8 (F, 46).

Some participants had been signposted to post-diagnostic support, however this had been designed for autistic people with high support needs or a learning disability, and was therefore not suitable for their needs [*“if there is any support available it is very much geared towards people with much higher support needs in terms of day to day living.” –* Participant 8 (F, 46)]. Some participants reported being perceived as too “high functioning” to require support (*“I said ‘what support is there?’ and they said ‘there isn’t any, because you are high functioning”’* – Participant 4 (M, 63). Services did not offer support to build understanding of the diagnosis, how it fits within a person’s identity, and build strategies to help them move forward.

*“Getting a diagnosis was also a frustrating thing because [*…*] there wasn’t really anything in the report that I was given at my diagnosis or after my diagnosis that really said anything like ‘well this is how it seems to us how your autism has affected you’.*” Participant 5 (M, 50).

Participants also reported barriers to accessing autism services. Some were oversubscribed with long waiting lists, so support was significantly delayed and not available when most needed (“*I had to wait a year for the post diagnostic group to start again. When I went along to that, only two of us turned up so they cancelled it so then I had to wait another four months until they set up another one, I was in crisis at that point and I really needed the support right then and there. Not*… *wait a few months*… *and then wait a year*… *it wasn’t great”* – Participant 3 (F, 31). Some faced geographical barriers due to living a long distance from services, especially those who did not live in cities [“*I found post diagnosis support tends to be concentrated in cities in Scotland, which is not very useful if you don’t live in those cities. Post diagnosis support should be throughout all towns and cities. I think that is why kind of models like peer support would be better*…*it would be easier to support people that way” –* Participant 7 (F, 43)]. Some found that communication difficulties related to being autistic made it difficult to reach out to services. Referrals had also been made to services with insecure funding, and the lack of consistent or sustainable long-term funding increased anxiety when engaging with a service, which in some cases led to reduced engagement.

*“The adult autism team are responsible for post diagnostic support but because of funding, I think it is just a four week programme now. And then that is it. You have had your support, that is all we have got.”* – Participant 3 (F, 31).

Some participants also found that receiving an autism diagnosis resulted in them losing support from health providers or third sector organisations, as it was assumed that their support was not suitable for an autistic person.

*“I asked our (local NHS) counselling service, I wanted to talk to somebody about stuff, but because I asked them ‘I want some help talking through stuff in relation to my autism diagnosis’ they said ‘oh no, we can’t help you, we are not specialist enough for that.”’ –* Participant 11 (F, 39).

*“I did phone mental health services and ask them for help. and they said autism isn’t a mental health issue. Even though that was where I got diagnosed and that I sounded quite articulate and I would be all right.” –* Participant 4 (M, 63).

In general, participants said that the most beneficial support to them would have included building a sense of identity and community, as well as self-care skills and tools to manage stress and sensory and social overload [“Figuring out what your autism is; what your sensory issues are; where you should avoid communication, things like that.” – Participant 7 (F, 43)].

Explaining what autism is to family, friends, and employers, and identifying and requesting beneficial adjustments could be difficult. Some participants highlighted that friends and family had difficulty in accepting and understanding their diagnosis, and it was particularly difficult when others doubted or disbelieved their diagnosis, or perceived them as so “high functioning” to not require adjustments.

*“I needed help explaining it to my family. Cause even though my mum was there (at the diagnosis) and got it explained to her, she hadn’t quite*… *she knew what autism was but she didn’t really know what it was in relation to me.”* – Participant 3 (F, 31).

*“I think people who weren’t autistic didn’t understand why it was important” –* Participant 6 (F, 30).

#### Subtheme 3: Processing and Reframing

All participants described their difficulties processing the diagnosis, and reframing history in light of their diagnosis. Though some highlighted that diagnosis does not change who you are as a person and that you are still the same person afterward, it is still an adjustment and a lot of information to process, and it can take a long time to get used to.

*“I guess it was*… *well it still is, a bit of a rollercoaster because you have to*… *it kind of forces you to look back on your life through a different lens. Lots of stuff made sense. There was a lot of positives, where I could look back and think*… *well actually I didn’t fail at this thing that I didn’t manage to do. There was a reason that I now understand so I can forgive myself for not going to a top university and becoming a high-flying lawyer or whatever. Because that just wasn’t the right thing. And now I understand why I struggled with certain things. So that made sense.*” – Participant 8 (F, 46).

*“I think maybe some kind of support to just sort of deal with the transition into sort of knowing. I usually describe it like if you had your skirt caught in your knickers or something and you realised in that moment and you are like ‘oh my god, it has been like this the whole time’ and you think back to all these social situations that you thought you were so dead on with and then you look back and you were like ‘no’.”* – Participant 6 (F, 30).

Participants experienced many emotions in the aftermath of diagnosis, including fear, happiness, validation, relief, upset, and anger, but lacked an understanding person to discuss this with. *“Initially you had this bounce of*… *you have this sort of elation of knowing what things are, and being able to reframe things, but actually the problems were still there. But then actually once that has subsided then it is still the ‘oh god, what do I do now?’ sort of a feeling.”* – Participant 9 (M, 36).

Prior to their diagnosis, participants had often experienced difficulties in their social life, complex sensory processing differences, and difficulties with executive function. Many participants felt an important part of the post-diagnostic process was to develop self-compassion and understanding of what being autistic means, and reframing their identity and expectations around them being an autistic person, rather than a “failed neurotypical.” Spending time with other autistic people in a peer support setting may have been particularly beneficial in understanding oneself and validating their experiences.

*“I would have been very happy to just to be in a room with people who had that new diagnosis in common*… *because I have found that in trying to understand how the course of my life has run, in the light of this new diagnosis I have very often figured things out on the basis of something that somebody else has said about the course of their life. And it has not always been*… *‘oh that has happened to me too’. It has sometimes been ‘oh well that didn’t happen to me’ or ‘I don’t have that particular expression of the condition so*… *how does it work in me instead?’. But it has been that prompt of having somebody else’s life story, if you like, that has been really important for me understanding myself I suppose, ultimately.” –* Participant 5 (M, 50).

*“Just having someone kind of validate your symptoms and what you have been through, and when you have been in that mindset of ‘no it is just me, I am just not coping well with this’. Suddenly going from seeing myself as like a slightly defective neurotypical person, to actually a pretty well coping autistic person.”* Participant 11 (F, 39).

#### Subtheme 4: Informal Support

In the absence of the formal support desired by autistic adults post-diagnosis, many participants reported turning to social media groups to connect with other autistic people [*“Most of the support I get is through social media*…*there is no real official diagnosis support”* – Participant 7 (F, 43)/“*Through twitter and facebook, I have found a community in which I get support. But when it comes to actual formal support in healthcare, it is a dead end for me”* – Participant 9 (M, 36)]. They noted how useful it could be to learn about autism from other autistic people [“*Through social media platforms, there are a lot of autistic advocates having their voice heard, which is absolutely fantastic, connecting with each other, and it has been invaluable to me”* – Participant 10 (F, 33)]. However, not all participants enjoyed social media engagement (“*Twitter, I found, fed a lot of my anxieties. And while you could meet people on it, it didn’t feel like an actual connection with people” –* Participant 6 (F, 30). *“Some people are very angry on there [social media] or really struggling and sometimes it can be quite depressing to read those things”* – Participant 2 (NB, 48), and thought they would have benefitted from in-person peer support [*“I got a lot of (autistic peer) support online, but I think I would have benefitted from in-person support, a similar thing. Just some place where I could speak to and meet other autistic people”* – Participant 7 (F, 43)].

Though research has shown the benefits of community engagement for autistic people, no participants mentioned being told this during the diagnostic process.

### Theme 2: Community Connection

The second theme focussed on community connection. Following their diagnosis, participants felt that they wanted to connect and engage with a new community of people, build connections with others who may have similar experiences to them, and that this may increase self-acceptance in being autistic.

#### Subtheme 1: Building Connections

Participants felt that after receiving an autism diagnosis, building connections with like-minded peers, and feeling part of a community was particularly important. Participants said that relationships with other autistic people felt easier and more comfortable than relationships with non-autistic people, and that they experienced feelings of similarity and connection with them. Participants felt that they were more able to create authentic connections with other autistic people because they had a reduced need to mask their natural autistic behaviours [*“It is great to have people who don’t need an explanation*… *you don’t need to hide your autistic traits*…*you know there is no need to worry with them.”* – Participant 7 (F, 43)]. This communicative ease and mutual understanding may create a comfortable and supportive environment for peer support following diagnosis, and more so than support provided by non-autistic people.

*“Autistic (peer support) would be the most beneficial. Just because you can never explain to someone who has never been othered, you can never explain what that feels like from birth. The level of personal reflection, doubt, fear, that that brings, whatever intersection you belong to. If you don’t belong to any, you can’t explain what that feels like, and the effect it has 30 years down the line, you know”* – Participant 10 (F, 33).

*“It just helps to have a group of fellow travellers who understand” –* Participant 8 (F, 46).

#### Subtheme 2: Identity and Visibility

Many participants reported that it took some time to overcome embedded internalised stigma, and that it was difficult to come to terms with some aspects of being autistic that they struggled with.

*In the past I really would have beaten myself up about it thinking*… *‘I need to go to these things’ and then I would go along to it, probably have to leave early and then feel like I had disappointed everybody and people would be angry with me and I would be angry with myself. So*… *I now think understanding that my brain works a bit differently in certain aspects is ok. And it is ok to be me and that I should not criticise myself for being me. –* Participant 1 (F, 54).

Participants felt that being around other autistic people, sharing mutual experiences and discussing challenges helped enhance their self-understanding and reduce internalised stigma. Peer support may provide a space where autistic adults can be comfortable in who they are, and discuss things they struggle with, with peers who have similar experiences.

*“Just having some people to say ‘this happens to me, is this normal? What do you do about this?’ is so important”* – Participant 11 (F, 39).

#### Subtheme 3: Community Diversity

While participants generally found interacting with autistic people more comfortable than interacting with non-autistic people, this is not a universal experience. All autistic people are different, and it cannot be assumed that all autistic people will experience similarities or have easier communication on the basis that they are autistic.

“*I think it is untrue, the idea that all autistic people get along with each other. They don’t. There is a heck of a lot of arguing going on on twitter amongst autistic people*…*All autistic people are different, even if there are some similarities by virtue of having the same diagnosis, and there will be some people who you get along with and some people you don’t”* – Participant 1 (F, 54).

Autistic people are not solely defined by being autistic and for peer support to be successful, participants felt it was important to take into account other aspects of their backgrounds [*“I find I am just as defined as being a carer for my husband and being a mother to my children than I am my autistic diagnosis. So those other roles need to be there, for them to truly understand.”* – Participant 10 (F, 33)]. It is also important to consider compatibility (or incompatibility) of communication styles and preferences of group members.

*“Just kind of finding compatible people. There is quite a few people who are very similar to me. We get on really well. Cause we are all quite quiet. We will talk if we are interested in something. But we won’t just talk and talk and talk and we will all be fine in a group. But as soon as you introduce someone who is louder, they kind of take over the whole group. Or somebody who can’t control the volume of their voice or whatever. Then you might get people going ‘shut up’. People who are more outspoken than me will then get annoyed and it will turn up into just a big barny, or argument and I am just like fleeing this issue. So it is hard to kind of*… *having that empathy for understanding people can’t help how they are.”* Participant 3 (F, 31).

### Theme 3: Flexible and Personalised Support

The third theme focussed on the need for flexible and personalised support. Participants reported needing a variety of support following the diagnosis, focussing on different areas, with different support intensity and length. Participants felt that peer support may offer the flexibility needed to improve their post-diagnostic experience.

#### Subtheme 1: Focus of Support

Peer support offers a flexible way to meet the needs of autistic people, which may vary both between people and over time. Participants reported that peer support could have a number of purposes, including sharing skills and strategies, providing a space to talk and socialise, or engaging in shared activities.

*“I suppose it is what they struggle with in particular. So some people really struggle with anxiety and depression and other people might struggle with socialising, someone else might struggle with sensory things. So there is different things that we struggle with. So maybe thinking about what area they would need support with.” –* Participant 2 (NB, 48).

Participants felt it was important that the focus of support should be led by those engaging with the peer support group at any given time, and so peer support frameworks should be flexible and responsive to the needs of group members.

#### Subtheme 2: Practicalities of Support

Preferences regarding the structure of peer support varied between participants, reflecting a variety of opinions. Peer support facilitators should be sensitive to the preferences of group members regarding group size (i.e., 1:1, group support, or mentoring), frequency of support (weekly, fortnightly, or monthly), and location (online or offline, and if online, the type of space used).

Peer support facilitators should ensure accessibility of spaces for autistic people, including considering the sensory environment, including photos and bios of facilitators, and a clear explanation of the roles of peer support facilitators.

*“In my area there is*… *another adult who I know through a friend, who is autistic, has said ‘oh there is this pub meet up for kind of Asperger’s type people’. It is like going to a pub to meet a load of people you don’t know, is like my worst nightmare. So I have never done it. It is just ironic, there is support there, but in order to access it you have to do the thing that is really difficult.” –* Participant 11 (F, 39).

*“Trust would be the biggest barrier to overcome for me. I would say that maybe a ‘get to know the mentors coffee morning’ so you can see which ones you get along with or maybe, an online bio and a photo, so you get a better grasp of who they are and what they are about, before you start trusting in people. Because post diagnosis you are very raw*… *well I was, and quite guarded about ‘well everybody has failed me’, not that that was true, but that was what it felt like.”* – Participant 10 (F, 33).

### Theme 4: Sustainability of Support

The final theme focussed on the sustainability of support. Participants expressed a need for peer support to be manageable and sustainable in the long term. Some participants expressed distress that they had engaged with formal or informal peer support groups, and had found that the group had been discontinued due to a lack of funding, space, or someone to co-ordinate.

*“I went to a sort of support group. Run by our local*… *psychiatric hospital, run by a chap from there. Which I already knew about and which I joined in as much as I could. But that has collapsed*…*because of funding”* – Participant 12 (M, 66).

#### Subtheme 1: Supporting Peer Support Facilitators

Simply being autistic does not provide someone with the knowledge and skills to be a peer support co-ordinator. Participants felt that peer support coordinators or mentors needed to have some training or experience, otherwise peer support may not function.

*“some kind of training on how*… *how to make sure [peer support coordinators] are communicating effectively to the particular person that they are mentoring*…*you need someone who can communicate with you in a way you respond to positively. Otherwise you are just going to feel even more alientated: here I am with this new diagnosis and even other “Aspys” or people on the spectrum don’t get me either”* – Participant 5 (M, 50).

Participants stated that peer support coordinators require training and support in order to be able to run a peer support service that meets the needs of a wide range of autistic adults.

*“If you are going to mentor someone you would need to tell them what other support is available. Training on what they are entitled to, what issues they might experience, where to get help, that sort of thing. And where to get help if they are having mental health problems or bullying at work, or anything like that. Some kind of psychological training or coaching because you don’t want to say the wrong thing”* – Participant 7 (F, 43).

*“Because they [peer support coordinators] are offering psychological support, they will need training before doing it.” –* Participant 8 (F, 46).

*“Training on group dynamics if they are not managed well, if there is a personality clash or something like that, places of inclusion can become quite exclusive”* Participant 9 (M, 36).

“*Some awareness of being a parent or carer*…*and I don’t know if they would have to go on a course to educate themselves but there are other things that non-white autistic people are party to that white autistic people wont understand*…*little bits of racism” –* Participant 10 (F, 33).

Additionally, participants reported that facilitators should have professional supervision to reduce the risk of burnout, and to enhance resilience and confidence.

*I think kind of like the supervision model is always a really good one. If you are being the person that is taking an emotional burden from somebody then you need somewhere to put it as well. So I think kind of having a forum where I would then be able to*… *either I had a mentor or there was some sort of group where mentors were able to chat stuff through and put stuff down, something like that would be really useful.* – Participant 11 (F, 39).

*“Being a peer support worker, there needs to be thoughts about workplace mental health, stress at work, how that affects you, the boundaries of it*…*I guess proper like supervision, where you were able to talk about these specific issues, that would be a massive part of it”* – Participant 6 (F, 30).

#### Subtheme 2: Providing Sustainable Infrastructure

Participants reported that long-term secure funding was essential for providing a stable environment for them to feel comfortable in the peer support relationship. Some participants had engaged with peer support or autistic-led support after their diagnosis, but a lack of sustainable funding had resulted in anxiety around whether support would be available to them beyond the short-term.

Providing administrative support to organise and host peer support is essential to ensure smoothly-run and consistent support: “…*it should be properly constituted, and perhaps even having some sort of professional workers there as facilitators who are there just to*… *you know manage the group and maybe the admin and booking rooms and things like that” –* Participant 9 (M, 36).

#### Subtheme 3: Working With Other Specialist Services

Many participants reported that following a diagnosis of autism, services were less likely to offer them support or be willing to provide them with care.

*“All of a sudden*… *IAPT and local community mental health services wouldn’t actually take a referral because they said it wasn’t within their expertise and that I should access the specialist service. But the specialist service was only a diagnostic service, not a general health and wellbeing and ongoing support service. So it actually closed things off.”* – Participant 9 (M, 36).

Additionally, some services did not consider the autism diagnosis and gave advice that was difficult to understand or apply.

*“One thing I have found with my chronic fatigue syndrome for example, some of the things I am expected to do for that are completely at 180 degrees from the things I am supposed to do to manage my depression, so it is very difficult to know what to do. Then when you add autism into the mix as well. It is just a further complication*…*that has been very, very frustrating and there has been absolutely no support at any level, from the state sector, the NHS, or from the third sector, on trying to figure that out.”* – Participant 5 (M, 50).

Participants noted the importance of recognising the boundaries and limitations of peer support, and acknowledged that autistic adults may require support from other healthcare providers. This may include accessing mental health care from specialist providers. Peer support frameworks should not be used in place of specialist support: it should work alongside specialist teams to ensure that autistic adults can access the support that best suits their needs.

## Discussion

In general, participants were very positive about the concept of post-diagnostic peer support. They felt it offered unique opportunities to engage with other autistic people, learn more about autism, and understand how their new diagnosis applied to their lives in a more meaningful way than the facts-based information that had been provided to them at their diagnosis. Participants liked the flexible nature of peer support and the opportunity to focus on a wide range of topics from an autistic perspective. Participants provided key insights into what peer support should focus on, how it should be run, and the specific benefits it may offer. According to these findings, maximising the potential of peer support will involve sustained funding, engaging with other specialist services, and training and support for facilitators. These findings are a crucial step in future studies that may examine the efficacy of a post-diagnostic peer support intervention for autistic adults.

Many of the findings of this research are supported by recent literature. One of the benefits of peer support indicated by this study is the relational and emotional benefits of autistic-autistic interaction. Being with other autistic people may act as a buffer to the effects of minority stress (Botha, 2020), and being around other autistic people in the post-diagnostic period may be especially beneficial (Crane et al., 2020). Alongside lived experience, autistic adults have fairly high levels of scientifically-based knowledge of autism (Gillespie-Lynch et al., 2017), and frame autism in a way that is embodied and positive (Welch et al., 2020). The need for post-diagnostic support that meets emotional and relational needs (as well as informational) has been also emphasised in the literature, though in the context of supporting parents after a child’s diagnosis (Legg and Tickle, 2019). Our results suggested similarly that a relational and emotional approach can be beneficial for autistic adults to help them re-frame their past experiences and work out where an autism diagnosis fits into their life. Providing a space for autistic people to interact and support one another in a comfortable way may also help build resilience to manage everyday life in a world that is designed for the non-autistic majority (Crompton et al., 2020a). This manuscript is among the first to explicitly examine how post-diagnostic peer support may function for autistic adults, exploring the views, preferences, and priorities of autistic adults relating to post-diagnostic peer support. Currently, many health and support services do not know what autistic people need (Doherty et al., 2020). This manuscript provides key insights from autistic people and paves the way for their voices to be centred in creating services that meet the needs of autistic people. By examining what the function and focus of peer support should be, future studies may in turn be able to focus on the efficacy of a post-diagnostic support intervention that is informed by these views.

### Practical Implications and Peer Support Design

It is essential for autistic people to be integrally involved with creating, designing, and implementing supports to ensure that they meet their needs (Monahan et al., 2021), and this study provides key grounding in post-diagnostic peer support design. Firstly, good communication is essential to providing peer support. Many participants were not able to access support due to inaccessible referral routes, poor communication while support was provided, or support starting or stopping with short or no notice. When accessing any new support, flexible communication is crucially important and this is no different for peer support. Participants stated a preference for support that focuses on progressing and re-framing during the post-diagnosis phase, rather than being based on facts about autism. While learning about autism was important, participants felt that it would be most helpful to have a chance to contextualise that information, and reflect on how their new diagnosis fit with their past experiences. Participants also stated that peer support facilitators require training in order to run a peer support service that meets the needs of a wide range of autistic adults, and that facilitators require ongoing support and supervision. To ensure that peer support is accessible, participants felt it was important that peer support frameworks take into account the diversity of the autistic population, the intersectionality of autism with other identities, and the communication preferences of group members. Participants felt it was essential that peer support infrastructure was sustainable with steady, long-term funding, and that engaging with other specialist services (for example mental health services) was well supported. They felt these actions creates a peer support space that feels safe and comfortable for autistic people to engage with.

### Strengths and Limitations

This study has some limitations. First, the sample included 12 autistic adults. The number of participants for this study was selected before commencing the study and is in line with similar studies in this area (see Cresswell et al., 2019), and based on prior research suggesting this sample size is sufficient to achieve saturation (Guest et al., 2006; Hennink and Kaiser, 2021). While a moderate sample size is standard in qualitative research in order to allow for an in-depth exploration of individual experience, findings may not apply to all autistic people diagnosed in adulthood. We cannot be sure from these data that all autistic people will equally want or benefit from the same kind of peer support. For example, not all newly diagnosed autistic people will accept or openly identify with their “new identity” as an autistic person, and it is important to ensure that autistic people can access the post-diagnosis support that best suits their needs at that time. While our sample had good diversity in terms of gender, age, and length of time since diagnosis, all participants were White British or European. It is important to consider that the views of autistic people from other ethnic backgrounds may differ, and they may have specific support needs and preferences (Jones et al., 2020). Additionally, all participants were based in the United Kingdom: their experiences of services may be shaped by United Kingdom norms and findings may not apply to autistic people living outside the United Kingdom. Participants had a variety of prior exposure to peer support: some had no experience, some had experienced informal peer support, and some had engaged with formal peer support. While this variety of experiences meant a range of views were represented, it did mean that the participant sample was not consistent in whether they were speaking from experience or speaking speculatively about peer support. We used a range of options for engaging with interviews to diversify the participant sample, including face-to-face, online messaging, video calls and phone calls. However, the sample is, of course, self-selecting and we may have over-representation of participants with an interest in autistic community and peer engagement.

## Conclusion

This study found that peer support may be a useful mechanism to support autistic adults after their diagnosis. Autistic adults were generally positive about the concept of peer support and the opportunity it provides to interact with others in a comfortable way, and to discuss difficulties with empathetic and understanding others. Peer support offers unique opportunities not available through other support channels, and can run alongside other specialist support if required. Peer support may be a sustainable and low cost option to fill the much-needed post-diagnostic support gap currently faced by autistic adults; however, careful planning, ongoing support and training for peer support facilitators, and centring the voices of autistic adults is essential to ensure the success of peer support programmes.

## Data Availability Statement

The datasets presented in this article are not readily available because of the potentially identifiable nature of the raw qualitative interview data. Requests to access the datasets should be directed to CC, catherine.crompton@ed.ac.uk.

## Ethics Statement

The studies involving human participants were reviewed and approved by University of Edinburgh Moray House School of Education Research Ethics Committee. The participants provided written informed consent to participate in this study.

## Author Contributions

CC: conceptualisation, funding acquisition, data collection and curation, formal analysis, project administration, and writing-original draft preparation. SH: formal analysis and writing-review and editing. CM: writing-original draft preparation. AS and SF-W: conceptualisation, funding acquisition, supervision, and writing-review and editing. All authors contributed to the article and approved the submitted version.

## Conflict of Interest

The authors declare that the research was conducted in the absence of any commercial or financial relationships that could be construed as a potential conflict of interest.

## Publisher’s Note

All claims expressed in this article are solely those of the authors and do not necessarily represent those of their affiliated organizations, or those of the publisher, the editors and the reviewers. Any product that may be evaluated in this article, or claim that may be made by its manufacturer, is not guaranteed or endorsed by the publisher.

## References

[B1] Appel K. (2011). “College mentoring programs,” in *Collaboration: A Multidisciplinary Approach to Educating Students With Disabilities*, 1st Edn, eds Simpson C. Bakken J. (Waco, TX: Prufrock Press Inc), 353–361.

[B2] Arnold S. R. Huang Y. Hwang Y. I. Richdale A. L. Trollor J. N. Lawson L. P. (2020). “The single most important thing that has happened to me in my life”: development of the impact of diagnosis scale—preliminary revision. *Autism Adulthood* 2 34–41. 10.1089/aut.2019.0059 PMC8992847 36600983

[B3] Bargiela S. Steward R. Mandy W. (2016). The experiences of late-diagnosed women with autism spectrum conditions: an investigation of the female autism phenotype. *J. Autism Dev. Disord.* 46 3281–3294. 10.1007/s10803-016-2872-8 27457364 PMC5040731

[B4] Baron-Cohen S. Wheelwright S. Skinner R. Martin J. Clubley E. (2001). The autism-spectrum quotient (AQ): evidence from asperger syndrome/high-functioning autism, malesand females, scientists and mathematicians. *J. Autism Dev. Disord.* 31 5–17. 10.1023/a:1005653411471 11439754

[B5] Barriball K. L. While A. (1994). Collecting data using a semi-structured interview: a discussion paper. *J. Adv. Nurs. Inst. Subscription* 19 328–335. 10.1111/j.1365-2648.1994.tb01088.x 8188965

[B6] Beresford B. A. Mukherjee S. K. M. Heavey E. (2019). “Adults’ experiences of the diagnostic assessment: what does it tell us about post-diagnostic support?,” in *Paper Presented at Autism–Europe International Congress*, (Nice).

[B7] Botha M. (2020). *Autistic Community Connectedness as a Buffer Against the Effects of Minority Stress.* Doctoral dissertation. Guildford: University of Surrey.

[B8] Bradley R. (2016). ‘Why single me out?’Peer mentoring, autism and inclusion in mainstream secondary schools. *Br. J. Spec. Educ.* 43 272–288.

[B9] Braun V. Clarke V. (2006). Using thematic analysis in psychology. *Qual. Res. psychol.* 3 77–101. 10.1191/1478088706qp063oa

[B10] Braun V. Clarke V. (2021). Can I use TA? Should I use TA? Should I not use TA? Comparing reflexive thematic analysis and other pattern-based qualitative analytic approaches. *Couns. Psychother. Res.* 21 37–47. 10.1002/capr.12360

[B11] Cage E. Di Monaco J. Newell V. (2018). Experiences of autism acceptance and mental health in autistic adults. *J. Autism Dev. Disord.* 48 473–484. 10.1007/s10803-017-3342-7 29071566 PMC5807490

[B12] Cassidy S. Bradley L. Shaw R. Baron-Cohen S. (2018). Risk markers for suicidality in autistic adults. *Mol. Autism* 9:42. 10.1186/s13229-018-0226-4 30083306 PMC6069847

[B13] Corden K. Brewer R. Cage E. (2021). Personal identity after an autism diagnosis: relationships with self-esteem, mental wellbeing and diagnostic timing. *PsyArXiv* [Preprint] 10.3389/fpsyg.2021.699335 PMC8360844 34393933

[B14] Crane L. Batty R. Adeyinka H. Goddard L. Henry L. A. Hill E. L. (2018). Autism diagnosis in the United Kingdom: perspectives of autistic adults, parents and professionals. *J. Autism Dev. Disord.* 48 3761–3772. 10.1007/s10803-018-3639-1 29948530 PMC6182717

[B15] Crane L. Hearst C. Ashworth M. Davies J. Hill E. L. (2020). Supporting newly identified or diagnosed autistic adults: an initial evaluation of an autistic-led programme. *J. Autism Dev. Disord.* 51 892–905. 10.1007/s10803-020-04486-4 32266684 PMC7954714

[B16] Cresswell L. Hinch R. Cage E. (2019). The experiences of peer relationships amongst autistic adolescents: a systematic review of the qualitative evidence. *Res. Autism Spectr. Disord.* 61, 45–60.

[B17] Crisp G. Cruz I. (2009). Mentoring college students: a critical review of the literature between 1990 and 2007. *Res. High. Educ.* 50 525–545. 10.1007/s11162-009-9130-2

[B18] Crompton C. J. Hallett S. Ropar D. Flynn E. Fletcher-Watson S. (2020a). ‘I never realised everybody felt as happy as I do when I am around autistic people’: a thematic analysis of autistic adults’ relationships with autistic and neurotypical friends and family. *Autism* 24 1438–1448. 10.1177/1362361320908976 32148068 PMC7376620

[B19] Crompton C. J. Ropar D. Evans-Williams C. V. Flynn E. G. Fletcher-Watson S. (2020b). Autistic peer-to-peer information transfer is highly effective. *Autism* 24 1704–1712. 10.1177/1362361320919286 32431157 PMC7545656

[B20] Crompton C. J. Sharp M. Axbey H. Fletcher-Watson S. Flynn E. G. Ropar D. (2020c). Neurotype-matching, but not being autistic, influences self and observer ratings of interpersonal rapport. *Front. Psychol.* 11:586171. 10.3389/fpsyg.2020.586171 33192918 PMC7645034

[B21] Davies B. (1996). *Volunteering Versus Professionalism: National Youth Agency Occasional Paper.* Leicester: National Youth Agency.

[B22] Doherty M. Neilson S. D.’Sullivan J. D. O. Carravallah L. Johnson M. Cullen W. (2020). Barriers to healthcare for autistic adults: consequences and policy implications. a cross-sectional study. *MedRxiv.*[preprint] 10.1101/2020.04.01.20050336

[B23] Fombonne E. (2005). The changing epidemiology of autism. *J. Appl. Res. Intellect. Disabil.* 18 281–294.

[B24] Geurts H. M. Jansen M. D. (2012). A retrospective chart study: the pathway to a diagnosis for adults referred for ASD assessment. *Autism* 16 299–305. 10.1177/1362361311421775 21949003

[B25] Gillespie-Lynch K. Kapp S. K. Brooks P. J. Pickens J. Schwartzman B. (2017). Whose expertise is it? Evidence for autistic adults as critical autism experts. *Front. Psychol.* 8:438. 10.3389/fpsyg.2017.00438 28400742 PMC5368186

[B26] Glaser B. G. Strauss A. L. (1967). *The Discovery of Grounded Theory: Strategies for Qualitative Research.* New York, NY: Aldine De Gruyter.

[B27] Gould J. Ashton-Smith J. (2011). Missed diagnosis or misdiagnosis? Girls and women on the autism spectrum. *Good Autism Pract. (GAP)* 12 34–41.

[B28] Griffith G. M. Totsika V. Nash S. Hastings R. P. (2012). ‘I just don’t fit anywhere’: support experiences and future support needs of individuals with asperger syndrome in middle adulthood. *Autism* 16 532–546. 10.1177/1362361311405223 21610188

[B29] Guest G. Bunce A. Johnson L. (2006). How many interviews are enough? An experiment with data saturation and variability. *Field Methods* 18 59–82.

[B30] Hansen S. N. Schendel D. E. Parner E. T. (2015). Explaining the increase in the prevalence of autism spectrum disorders: the proportion attributable to changes in reporting practices. *JAMA Pediatr.* 169 56–62. 10.1001/jamapediatrics.2014.1893 25365033

[B31] Happé F. G. Mansour H. Barrett P. Brown T. Abbott P. Charlton R. A. (2016). Demographic and cognitive profile of individuals seeking a diagnosis of autism spectrum disorder in adulthood. *J. Autism Dev. Disord.* 46 3469–3480. 10.1007/s10803-016-2886-2 27549589

[B32] Heasman B. Gillespie A. (2019). Neurodivergent intersubjectivity: distinctive features of how autistic people create shared understanding. *Autism* 23 910–921. 10.1177/1362361318785172 30073872 PMC6512057

[B33] Hennink M. Kaiser B. N. (2021). Sample sizes for saturation in qualitative research: a systematic review of empirical tests. *Soc. Sci. Med.* 292:114523. 10.1016/j.socscimed.2021.114523 34785096

[B34] Hickey A. Crabtree J. Stott J. (2018). Suddenly the first fifty years of my life made sense’: experiences of older people with autism. *Autism* 22 357–367. 10.1177/1362361316680914 29153003

[B35] Hillier A. Goldstein J. Tornatore L. Byrne E. Johnson H. (2019). Outcomes of a peer mentoring program for university students with disabilities. *Mentor. Tutoring Partnership Partnersh. Learn.* 27 487–508. 10.1080/13611267.2019.1675850

[B36] Huang Y. Arnold S. R. Foley K. R. Trollor J. N. (2020). Diagnosis of autism in adulthood: a scoping review. *Autism* 24 1311–1327. 10.1177/1362361320903128 32106698

[B37] Jacobi M. (1991). Mentoring and undergraduate academic success: a literature review. *Rev. Educ. Res.* 61 505–532. 10.3102/00346543061004505

[B38] Jones D. R. Nicolaidis C. Ellwood L. J. Garcia A. Johnson K. R. Lopez K. (2020). An expert discussion on structural racism in autism research and practice. *Autism Adulthood* 2 273–281. 10.1089/aut.2020.29015.drj PMC8992862 36600959

[B39] Jones L. Goddard L. Hill E. L. Henry L. A. Crane L. (2014). Experiences of receiving a diagnosis of autism spectrum disorder: a survey of adults in the United Kingdom. *J. Autism Dev. Disord.* 44 3033–3044. 10.1007/s10803-014-2161-3 24915932

[B40] Leedham A. Thompson A. R. Smith R. Freeth M. (2020). ‘I was exhausted trying to figure it out’: the experiences of females receiving an autism diagnosis in middle to late adulthood. *Autism* 24 135–146. 10.1177/1362361319853442 31144507

[B41] Legg H. Tickle A. (2019). UK parents’ experiences of their child receiving a diagnosis of autism spectrum disorder: a systematic review of the qualitative evidence. *Autism* 23 1897–1910. 10.1177/1362361319841488 30995082

[B42] Lewis L. F. (2017). A mixed methods study of barriers to formal diagnosis of autism spectrum disorder in adults. *J. Autism Dev. Disord.* 47 2410–2424. 10.1007/s10803-017-3168-3 28516422

[B43] MacLeod A. (2010). ‘Welcome to my first rant!’report on a participatory pilot project to develop the ‘as portal’, an online peer support network for higher education students on the autism spectrum. *J. Assist. Technol.* 4 14–24.

[B44] Maitland C. A. Rhodes S. O’Hare A. Stewart M. E. (2021). Social identities and mental well-being in autistic adults. *Autism* 25 1771–1783. 10.1177/13623613211004328 34011188 PMC8323333

[B45] Martin N. Milton D. Sims T. Dawkins G. Baron-Cohen S. Mills R. (2017). Does “mentoring” offer effective support to autistic adults? A mixed-methods pilot study. *Adv. Autism* 3 229–239. 10.1108/aia-06-2017-0013

[B46] Matson J. L. Shoemaker M. (2009). Intellectual disability and its relationship to autism spectrum disorders. *Res. Dev. Disabil.* 30 1107–1114. 19604668 10.1016/j.ridd.2009.06.003

[B47] Miles M. B. Huberman A. M. (1994). *Qualitative Data Analysis: An Expanded Sourcebook.* Thousand oaks, CA: Sage.

[B48] Milton D. Sims T. (2016). How is a sense of well-being and belonging constructed in the accounts of autistic adults? *Disabil. Soc.* 31 520–534.

[B49] Monahan J. Freedman B. Pini K. Lloyd R. (2021). Autistic input in social skills interventions for young adults: a systematic review of the literature. *Rev. J. Autism Dev. Dis.* 1–21. 10.1007/s40489-021-00280-9

[B50] Murphy C. M. Wilson C. E. Robertson D. M. Ecker C. Daly E. M. Hammond N. (2016). Autism spectrum disorder in adults: diagnosis, management, and health services development. *Neuropsychiatr. Dis. Treat.* 12 1669–1686. 10.2147/NDT.S65455 27462160 PMC4940003

[B51] Pfeiffer P. N. Heisler M. Piette J. D. Rogers M. A. Valenstein M. (2011). Efficacy of peer support interventions for depression: a meta-analysis. *Gen. Hosp. Psychiatry* 33 29–36. 10.1016/j.genhosppsych.2010.10.002 21353125 PMC3052992

[B52] Punshon C. Skirrow P. Murphy G. (2009). The’ not guilty verdict’ psychological reactions to a diagnosis of asperger syndrome in adulthood. *Autism* 13 265–283. 10.1177/1362361309103795 19369388

[B53] Raymond-Barker P. Griffith G. M. Hastings R. P. (2018). Biographical disruption: experiences of mothers of adults assessed for autism spectrum disorder. *J. Intellect. Dev. Disabil.* 43 83–92. 10.3109/13668250.2016.1262011

[B54] Repper J. Carter T. A. (2011). Review of the literature on peer support in mental health services. *J. Ment. Health* 20 392–411. 21770786 10.3109/09638237.2011.583947

[B55] Rifai O. M. Fletcher-Watson S. Jiménez-Sánchez L. Crompton C. J. (2021). Investigating markers of rapport in autistic and neurotypical interactions. *Autism Adulthood*. 10.1089/aut.2021.0017 [Epub ahead of print]. PMC8992924 36600904

[B56] Rutherford M. McKenzie K. McClure I. Forsyth K. O’Hare A. McCartney D. (2016a). A national study to investigate the clinical use of standardised instruments in autism spectrum disorder assessment of children and adults in Scotland. *Res. Autism Spec. Disord.* 29 93–100. 10.1016/j.rasd.2016.05.003

[B57] Rutherford M. McKenzie K. Forsyth K. McCartney D. O’Hare A. McClure I. (2016b). Why are they waiting? Exploring professional perspectives and developing solutions to delayed diagnosis of autism spectrum disorder in adults and children. . *Res. Autism Spec. Disord.* 31 53–65. 10.1016/j.rasd.2016.06.004

[B58] Rutter M. (2005). Aetiology of autism: findings and questions. *J. Intellect. Disabil. Res.* 49 231–238. 10.1111/j.1365-2788.2005.00676.x 15816809

[B59] Saldaña J. (2021). *The Coding Manual for Qualitative Researchers.* Thousand oaks, CA: Sage.

[B60] Skirrow P. Farrington J. (2008). Developing specialist services for adults with Asperger syndrome in Liverpool. *Clin. Psychol. Forum* 185 15–20.

[B61] Smith R. Netto J. Gribble N. C. Falkmer M. (2021). ‘At the end of the day, it’s love’: an exploration of relationships in neurodiverse couples. *J. Autism Dev. Disord.* 51 3311–3321. 10.1007/s10803-020-04790-z 33216278

[B62] Stevenson K. Cornell K. Hinchcliffe V. (2016). ‘Let’s talk autism’ -a school-based project for students to explore and share their experiences of being autistic. *Support Learn.* 31 208–234. 10.1111/1467-9604.12130

[B63] Tan C. D. (2018). “I’m a normal autistic person, not an abnormal neurotypical”: autism spectrum disorder diagnosis as biographical illumination. *Soc. Sci. Med.* 197 161–167. 10.1016/j.socscimed.2017.12.008 29247898

[B64] Thompson C. McDonald J. Kidd T. Falkmer T. Bölte S. Girdler S. (2020). “I don’t want to be a patient”: peer mentoring partnership fosters communication for autistic university students. *Scand. J. Occup. Ther.* 27 625–640. 10.1080/11038128.2020.1738545 32180486

[B65] Welch C. Cameron D. Fitch M. Polatajko H. (2020). From “since” to “if”: using blogs to explore an insider-informed framing of autism. *Disabil. Soc.* 1–24.

[B66] Willig C. (2013). *Introducing Qualitative Research In Psychology.* New York, NY: McGraw-hill.

