# Supplementary material for: “A Group of Fellow Travellers Who Understand”: Interviews With Autistic People About Post-diagnostic Peer Support in Adulthood
Source: Front Psychol. 2022 Mar 7;13:831628. doi: 10.3389/fpsyg.2022.831628 (PMC8964394; doi:10.3389/fpsyg.2022.831628)
Supplement: Supplementary file 1 [file Table_1.DOCX]

**Supplementary Table 1: Interview Schedule**

| **A: I’m going to start by asking you some questions about when you were diagnosed as autistic.** | |
| --- | --- |
| 1 | How old were you when you were diagnosed? How long ago was that? What led to you seeking a diagnosis of autism? |
| 2 | Can you tell me a bit about what the diagnosis process involved? |
| 3 | How did you feel in the days and weeks immediately following your diagnosis? Immediately after your diagnosis, what kinds of things did you need help and support with? |
| 4 | What kind of support did you receive after your diagnosis? Do you think you got enough post-diagnostic support? |
| 5 | How did you feel a year on from your diagnosis? Have there been any significant changes in how you feel since then? |
| **B. I’d like to ask you about the people in your life who have had a positive impact on you, since your diagnosis.** | |
| 6 | Have you had people in your life who support and mentor you? This might involve giving advice, listening to you, helping you work through your options, learning together. Were they autistic or not? How has this person/these people helped you understand your identity? |
| 7 | Do you think that developing an understanding of yourself as an autistic person has impacted different areas of your life? |
| **C. I’d now like ask you about peer support.** | |
| 8 | Have you ever had autistic peer support? This doesn’t have to be part of a formal arrangement – it could be having an autistic friend or relative who is particularly helpful and open to listening and advising, or engaging in an online or real world autistic community? |
| 9 | Is there anything in particular that you think is unique about how autistic people could support each other? |
| 10 | Is there anything in particular that you think would be difficult or challenging around how autistic people could support each other.? |
| 11 | If you were to have an autistic mentor, an autistic person who could listen and mentor you, is there anything important to have in common with them*?* For example would you like someone the same age, or gender, someone with a similar education or employment background? Or something else? |
| 12 | If you would have wanted peer support, when would it have been most useful for you? For example, 2 weeks after your diagnosis, 3 months after diagnosis, a year after diagnosis? |
| 13 | If you were to be an autistic mentor, what would you need to be a successful mentor? What would you want to know? What would you tell a mentee? |
| **D. Closing.** | |
| 14 | Thank you for sharing your experiences with me. Do you have anything else that you want to ask, or is there anything I didn’t ask you that you would like to talk about? |
